# Effects of deprescribing from inhaled corticosteroids in people with cystic fibrosis: protocol for a target trial emulation using the UK CF Registry

**DOI:** 10.1136/bmjopen-2025-100894

**Published:** 2025-10-29

**Authors:** Elliot McClenaghan, Julie Rouette, Emily Granger, Gwyneth Davies, Ruth H Keogh, John Tazare

**Affiliations:** 1 Department of Medical Statistics, London School of Hygiene & Tropical Medicine, London, UK; 2 Global Epidemiology, Office of the Chief Medical Officer GSK R&D, GlaxoSmithKline Plc, Montreal, Quebec, Canada; 3 Population Policy and Practice Research and Teaching Department, University College London Great Ormond Street Institute of Child Health, London, UK

**Keywords:** Cystic fibrosis, EPIDEMIOLOGY, STATISTICS & RESEARCH METHODS

## Abstract

**Abstract:**

**Introduction:**

Observational data are increasingly used to study and draw causal inferences about the effects of treatments. Target trial emulation (TTE) is a framework for mitigating biases in causal investigations through specification of an observational study, targeting a specific causal research question, based on a real or hypothetical randomised controlled trial. Investigations into the effects of treatment discontinuation are of growing interest and particularly relevant in cystic fibrosis (CF), where treatment burden is high and new transformative therapies are becoming widespread. We aim to use the TTE framework to investigate the effect of discontinuation of inhaled corticosteroids (ICS) on clinical outcomes in people with CF. Our observational emulation will be based on the CF WISE (Withdrawal of Inhaled Steroids Evaluation) trial (PMID:16556691).

**Methods and analysis:**

Two study designs proposed for investigating treatment effects using observational data are the prevalent new-user design and the sequential trials design. Each design uses different but related methods to address similar causal questions; however, the comparability between them remains uncertain. We will conduct a population-based cohort study using data from the UK CF Registry between January 2016 and June 2018 and apply these designs. We will specify the target trial protocol for each study design. Estimates for the causal effects of discontinuing ICS will be obtained and compared with those from the CF-WISE trial.

**Ethics and dissemination:**

This study has received approval from the UK CF Registry Research Committee for both the research and access to data. Ethical approval has also been granted by the LSHTM Ethics Committee. The UK CF Registry has NHS Research Ethics Committee approval (REC reference: 24/EE/0012). The findings from this project will be submitted to peer-reviewed journals and presented at academic conferences.

**STRENGTHS AND LIMITATIONS OF THIS STUDY**

- This protocol describes the first real-world cohort study on treatment discontinuation in people with cystic fibrosis (CF).
- The analysis will use registry data with near-whole population coverage for people with CF in the UK.
- The study will compare two causal study designs—the prevalent new-user design and sequential trials design—under the target trial emulation framework, with findings benchmarked against a CF trial.
- Both study designs have been shown to mitigate measured confounding and avoidable design-related biases such as selection bias and immortal time bias.
- Limitations include potential for errors in treatment discontinuation dates and lack of information on adherence.

## Introduction

 The target trial emulation (TTE) framework has been proposed to identify and avoid potential biases when estimating the causal effects of treatment using observational data. The strategy entails specifying protocol components of a real or hypothetical randomised controlled trial (RCT) (the ‘target trial’), including the causal estimand of interest, and detailing the emulation of this using the available observational data. The framework has the potential to minimise common time-related biases in pharmacoepidemiology and provides an opportunity to validly address a breadth of research questions, including about comparative effectiveness and treatment discontinuation.^1^,^4^

TTE has been adopted in many therapeutic areas, including cystic fibrosis (CF).^5^,^7^ CF is a life-shortening condition, caused by mutations in the CF transmembrane conductance regulator (CFTR) gene, which affects multiple organ systems, including the lungs, liver, digestive tract and reproductive organs. There are over 11 000 people in the UK living with CF.^8 9^ People with CF often face substantial treatment burden, requiring complicated and time-consuming treatment plans daily to maintain their health.^10^ This includes multiple different daily oral and inhaled therapies on a long-term basis as an integral component of management for the majority of people with CF.

Inhaled corticosteroids (ICS) are commonly used by people with CF, with the purported aim of targeting inflammation and/or symptoms of wheeze. However, the current evidence for risks and benefits of ICS in CF is uncertain, with known adverse effects on growth and adrenal function.^11^ A Cochrane review of ICS effectiveness in people with CF concluded that none of the currently published trials could conclude effectiveness at reducing lung inflammation; despite this, ICS are still widely prescribed.^9^ The only trial included which was found to have a low risk of bias was the CF-WISE (Withdrawal of Inhaled Steroids Evaluation) trial (PMID:16556691).^11 12^ CF-WISE investigated the effect of discontinuing ICS on risk of pulmonary exacerbation in the UK and concluded that it was safe for most patients to discontinue.^12^

This study aims to estimate the causal effects of discontinuing ICS on clinical outcomes in people with CF using ICS, using observational data from the UK CF Registry to emulate a target trial based on the CF-WISE trial (the ‘index trial’). Studying treatment discontinuation effects in observational data is challenging, with a notable evidence gap around the optimal methodological approach. Using the TTE framework, we will therefore consider two distinct study designs—the prevalent new-user design (PNUD) and the sequential trials design—and empirically compare the results between these designs and the published CF-WISE trial findings.

This study will also provide a framework for further trial emulations in the contemporaneous era where there is now widespread use of CFTR modulator treatments which are disease modifying and transforming clinical outcomes.^8 13^ This will contribute to the evidence base for medication use for the CF community in a time of rapid change in the treatment landscape. It will provide insight into the relative strengths and weaknesses of the two approaches (PNUD and sequential trials design), which will inform the wider field of treatment deprescribing. This will contribute to better understanding and improved accessibility of causal study designs for treatment discontinuation, to support further application of these designs and the TTE framework, including questions not answered by RCTs such as the longer-term effects of ICS discontinuation.

This study protocol is written using the HARmonised Protocol Template to Enhance Reproducibility guidelines.^14^

## Research aim and objectives

### Research aim

We aim to estimate the causal effects of discontinuing ICS on clinical outcomes (pulmonary exacerbations and lung function) in people with CF using two different study designs in observational data.

### Objectives

- To emulate a target trial using UK CF Registry data based on a previously conducted RCT, CF-WISE and assess whether the findings of the emulations are consistent with the results from the CF-WISE trial.
- To apply the PNUD and sequential trials design to emulate a discontinuation trial, to establish the estimands being targeted under the two designs, compare the results and interpret any differences.

## Methods and analysis

### Data source

The UK CF Registry (UKCFR) is a centralised national database sponsored and managed by the CF Trust, which collects real-world data from all specialist CF centres in the UK.^15^ It holds data on an estimated 99% of the UK CF population covering demographic and longitudinal clinical data and was highlighted by the UK National Institute for Health and Care Excellence real-world evidence framework as an exemplar data source for evidence generation.^16^

Data collection for the UKCFR began in 1996 and is primarily structured around annual review visits, taking place approximately 1 year apart. In addition, demographic data including sex, ethnicity, diagnosis data and genotype are collected at enrolment into the Registry and usually remain time-invariant. Information is captured annually on events or conditions that have happened since the previous annual review, including complications, infections, treatments and hospital admissions. Other information collected at the date of each annual review includes results from pulmonary function tests, weight and height. Extracts of data recorded at annual reviews are provided to researchers for approved projects. In 2016 variable domains were added, including exact start and stop dates of medications.

### The index trial: CF-WISE

CF-WISE was a double-blind RCT investigating the safety of withdrawing ICS in people with CF.^12^ Participants were recruited from 18 UK CF centres and the trial was carried out between November 2001 and July 2004. Participants taking fluticasone propionate (a specific ICS) were randomised to either discontinuation or continued use and followed up for 6 months, with time to first pulmonary exacerbation as the primary outcome.^12^ There was no evidence of a change in time to first exacerbation with HR (95% CI) 1.07 (0.68 to 1.70) and no change in secondary outcomes for fluticasone propionate versus placebo and based on this, it was concluded likely safe for most patients to withdraw from the medication.^12^ Despite this, ICS use remains common in people with CF with 20% of adults in the UK being prescribed this treatment.^9^

### Study design

We will use UKCFR data to conduct an emulation of a target trial based on the CF-WISE trial. This will be done using two study designs: (1) the PNUD and (2) the sequential trials design.^3 17^ The two study designs use distinct but related approaches to making use of observational data and applying statistical analyses to estimate causal effects, with control for measured confounding and time-related biases being at both design and analysis phases.^3 17^ The data used for these emulations will be from 2016 to 2018, chosen intentionally based on data quality and completeness in the UKCFR data and being in the pre-CFTR modulators era. Table 1 specifies the protocol components of the index trial (CF-WISE), the corresponding target trial (hypothetical RCT we would undertake if it were feasible) and how each element of the target trial will be emulated using UKCFR data.

**Table 1:** Specification and emulation of a target trial of deprescribing from inhaled corticosteroids in the UKCFR

Protocol component	CF-WISE (index randomised trial*)	Target trial	Target trial emulation using UKCFR
Eligibility criteria	People diagnosed with CF aged ≥6 years, with FEV _1_≥40% predicted, sustained ICS use ≥3 months. Recruitment period November 2001 and July 2004. Excluded are people who used oral corticosteroids, high-dose ICS or intravenous antibiotics in the 4 weeks prior to treatment assignment.	Same as CF-WISE, except study period is between January 2016 and June 2018. This period reflects a balance between data quality and completeness in UKCFR and prior to the widespread introduction of CFTR modulators.	Prevalent new-user design*:* Same as target trial, except ICS dosage was not recorded. Individuals are eligible for the base cohort (see *Study design*) on the earliest date within the study period that they meet all eligibility criteria. Sequential trials design: Same as target trial, except ICS dosage not recorded. Successive regularly spaced ‘trials’, with eligibility criteria redefined based on most recent data if available.
Treatment strategies	Following a 2-month run-in period receiving fluticasone propionate, participants either: continue to take fluticasone, or switch to taking placebo.	Participants either: continue to take ICS (any ICS, not limited to fluticasone propionate) for the duration of follow-up, or discontinue ICS and remain not taking it for the duration of follow-up.	Prevalent new-user design: Same as target trial, with information on (dis)continuation obtained using prescription start and stop dates. Sequential trials design: Same as the PNUD.
Assignment procedures	Randomisation to treatment strategy Investigators and participants blinded to assignment.	Same as CF-WISE.	Prevalent new-user design: For each discontinuer, comparators will be selected into time-based exposure sets that correspond to each ICS discontinuer. Comparators within each exposure set will then be reweighted to resemble the discontinuers. Sequential trials design: Sequence of artificial ‘trials’, with discontinuers and continuers defined within each regularly spaced ‘trial’. Potential for re-use of continuers across multiple trials. Confounder adjustment is through regression. In the absence of randomisation, treatment assignment is assumed to be random conditional on ICS treatment history and the measured covariates specified in *Covariates*.
Follow-up and index date	Follow-up starts at randomisation and ends at loss to follow-up, meeting criteria for early trial withdrawal, or 6 months of follow-up, whichever occurs first.	Same as CF-WISE	Prevalent new-user design: Follow-up starts at date of ICS discontinuation for both the discontinuers and the reweighted continuers. Sequential trials design: Follow-up starts at the date of ICS discontinuation for the discontinuers and the date of the start of each successive ‘trial’ for the continuers in that trial. For the primary outcome, individuals will be followed up to first pulmonary exacerbation, death, transplant or 6 months after treatment assignment, whichever occurs first. For information on follow-up for secondary outcomes (lung function) see online supplemental material appendix 1.
Outcomes	Primary: time to first pulmonary exacerbation Secondary: (i) lung function change (FEV_1_ and FVC), (ii) new/unplanned courses of intravenous antibiotics and (iii) rescue bronchodilator usage	Same as CF-WISE	Primary: same as target trial, with different definition for pulmonary exacerbation; defined as a course of hospital or home intravenous antibiotics (see *Outcomes*). Secondary: (i) mean change in FEV_1_% predicted and FVC% predicted. Secondary outcomes (ii) and (iii) will not be assessed due to similarity of UKCFR definitions for intravenous antibiotic courses and pulmonary exacerbation and inability to define rescue bronchodilator use, respectively.
Causal contrasts	Intention-to-treat	Same as CF-WISE	Observational analogue of intention-to-treat and per-protocol
Statistical analysis	For primary outcome, a Cox proportional hazard regression was used. The Wilcoxon rank sum test was used to compare % change in lung function. ORs or relative risks were estimated to compare categorical outcomes (ii and iii). Prespecified subgroup analyses for age, atopic status, dose of ICS, baseline FEV_1_ and *Pseudomonas aeruginosa* infection status.	Same as CF-WISE	Prevalent new-user design: Weighted Cox proportional hazards regression will be used to estimate HRs and 95% CIs. Marginal survival curves and risk difference at 6 months will be estimated. Sequential trials design: Adjusted Cox proportional hazards regression to estimate HRs and 95% CIs. Marginal survival curves and risk difference at 6 months estimated using standardisation. For both designs, intention to treat and per-protocol estimates will be calculated, the latter using inverse probability of censoring weights to account for informative censoring where individuals switch from assigned treatment during follow-up. For information on analysis for secondary outcomes (lung function) see online supplemental material appendix 1 .

* When the target trial is based on an existing RCT this corresponding RCT is termed the reference or index trial. Some aspects of the index trial and target trial may differ because it is not possible to emulate them in observational data, and separating the index and target trials can help to highlight where any differences in findings from the emulated trial compared with the index trial may arise.^48 49^

CFTR, cystic fibrosis transmembrane conductance regulator; CF WISE, Cystic Fibrosis Withdrawal of Inhaled Steroids Evaluation; FEV1, forced expiratory volume in 1 s; FVC, forced vital capacity; ICS, inhaled corticosteroids; PNUD, prevalent new-user design; RCT, randomised controlled trial; UKCFR, UK Cystic Fibrosis Registry.

#### Prevalent new-user design

The PNUD extends the scope of inference of the incident new-user design to include switchers to a (typically new) study drug from an existing comparator drug (ie, prevalent new-users).^17 18^ Beyond the switching context, PNUD can be used to study causal questions surrounding treatment discontinuation; here, the drug discontinuer is conceptualised as the ‘prevalent new-user’ and the continuer is the comparator.^19 20^

**Figure 1:**
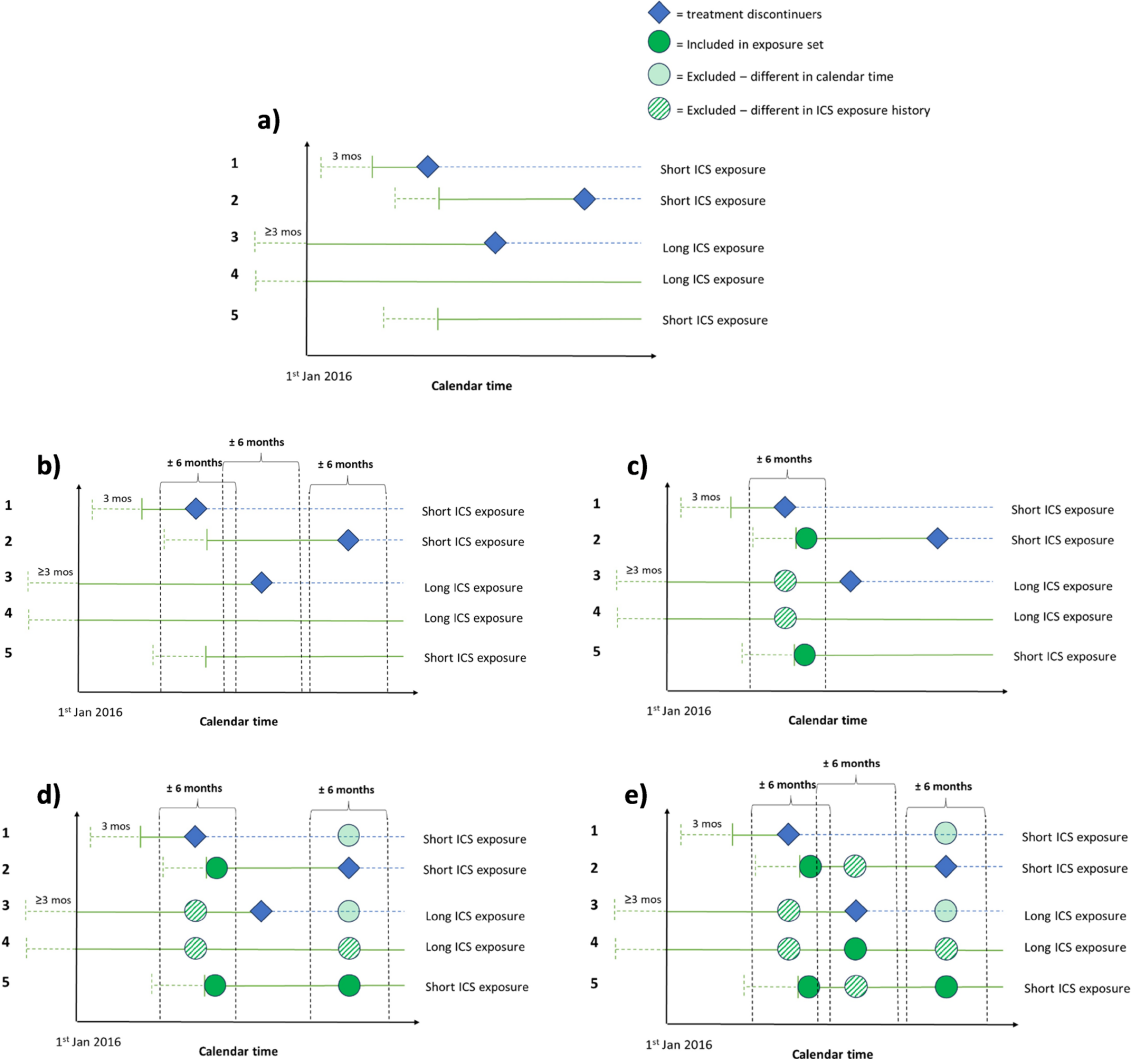
Study diagrams showing the selection of comparators and formation of analysis cohorts for emulating a target trial of discontinuation of inhaled corticosteroids (ICS) using the UK Cystic Fibrosis Registry (UKCFR), using the prevalent new-user design. Dotted green lines represent time an individual is taking ICS but not yet eligible for inclusion in the base cohort, solid green lines represent time an individual is taking ICS after more than 3 months of initial use, dotted blue lines represent time an individual is no longer taking ICS. (a) Five example individuals who meet the eligibility criteria for inclusion in the base cohort, where 1–3 discontinue ICS in the study period. (b) The criteria for being in each of the discontinuer’s exposure sets in calendar time (6 months either side date of discontinuation). (c) Individuals 2 and 5 are included in individual 1’s exposure set due to similarity in both calendar time and ICS exposure history. (d) Only individual 5 would be included in individual 2’s exposure set and (e) only individual 4 would be included in individual 3’s exposure set.

The first step of the PNUD will be to define a base cohort with respect to pre-defined eligibility criteria in the study period (see *Inclusion and exclusion criteria*, table 1). So-called ‘exposure sets’ will then be defined, each of which includes a discontinuer plus people remaining on ICS (continuers) who are similar to the discontinuer in terms of being observed within a similar calendar period and their ICS exposure history. Figures1  2 illustrate the formation of these exposure sets, each comprising a discontinuer and a pool of comparators (continuers). Treatment discontinuation (and continuation) will be determined based on treatment start and stop dates as recorded in the UKCFR. The analysis then involves reweighting continuers so that the comparators resemble the discontinuer with respect to measured baseline covariates (detailed in *Main analysis*).

**Figure 2:**
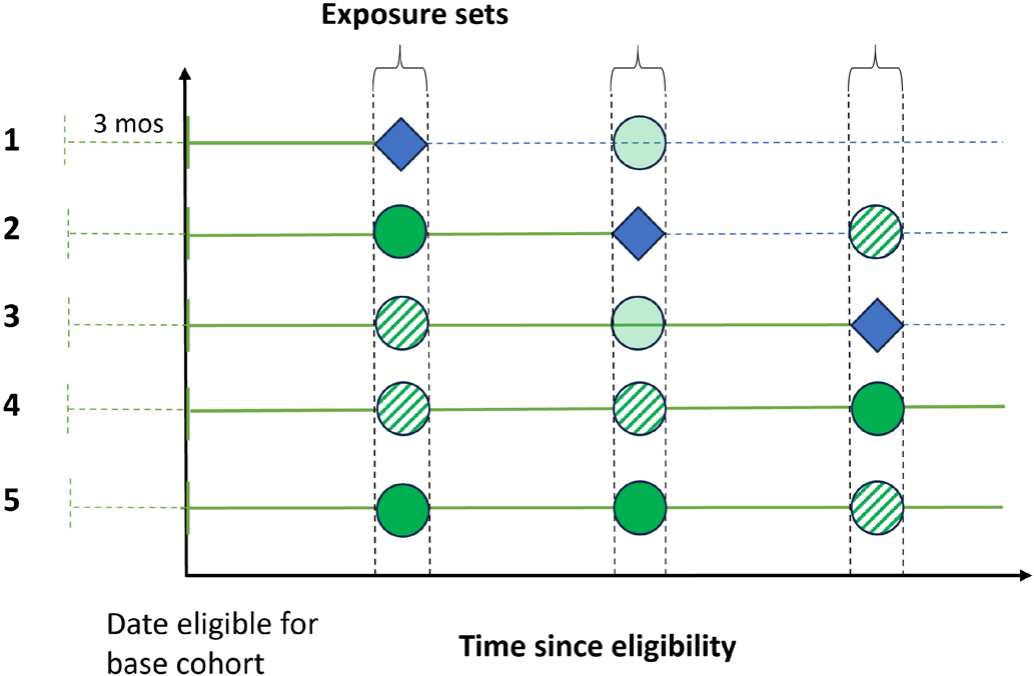
Study diagram showing the selection of comparators and formation of analysis cohorts for emulating a target trial of discontinuation of inhaled corticosteroids using the UK Cystic Fibrosis Registry (UKCFR), using the prevalent new-user design (PNUD) on an alternative time scale (time since eligibility). This diagram displays five example individuals who meet eligibility criteria for inclusion in the base cohort (the same as in figure 1), with the exposure sets on a time since eligibility time scale (a PNUD visualisation commonly used to display the organisation of prevalent new-users and comparators in the analysis cohort).^17^

#### Sequential trials design

**Figure 3:**
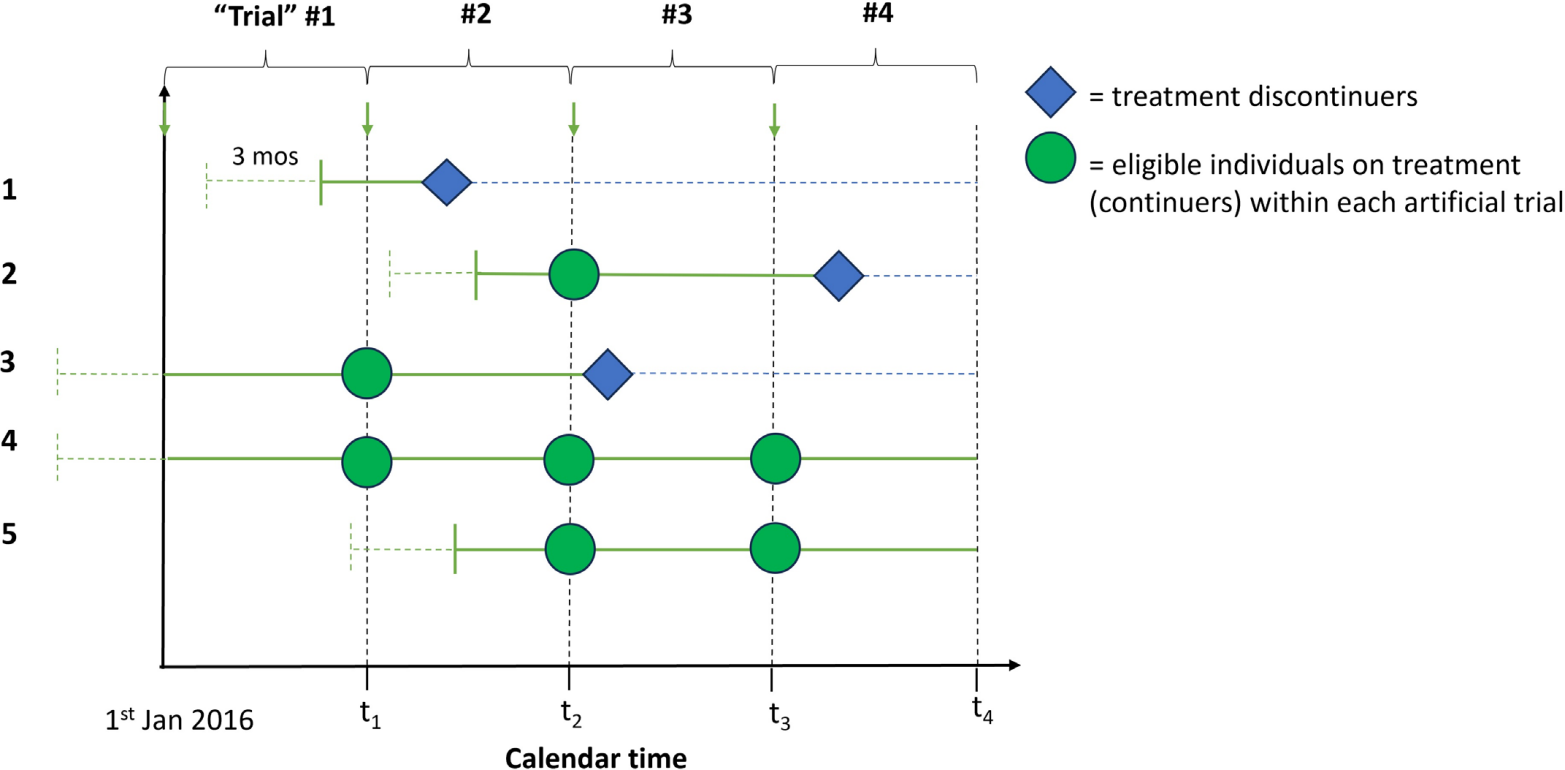
Study diagram showing the selection of comparators, artificial trials and formation of analysis cohorts for emulating a target trial of discontinuation of inhaled corticosteroids (ICS) using the UK Cystic Fibrosis Registry (UKCFR), using the sequential trials design on the calendar time scale. This diagram displays five example individuals (the same as in figures1  2) who meet eligibility criteria of a sequence of four artificial sequential trials in the study period. Within each artificial trial, individuals are separated into ICS discontinuers and continuers, with time zero being the date of discontinuation for discontinuers and the first calendar day of the trial for the continuers.

The sequential trials design is an alternative method which has elsewhere been referred to as the sequential trials approach, successive trials or sequential Cox approach.^20^,^22^ Under this design, a sequence of artificial ‘trials’ will commence at successive regularly spaced time points. At each time origin, the set of individuals meeting the eligibility criteria will be divided into ICS discontinuers and continuers based on treatment start and stop dates as recorded in the UKCFR, emulating a series of successive ‘trials’ from January 2016 to June 2018 (see figure 3). For example, this may entail successive monthly trials within which continuers and new discontinuers are identified. Eligible individuals who continue ICS may be included in multiple artificial trials. For example, individuals eligible for the trial commencing January 2016 but who do not discontinue ICS in that period will be eligible to be included in the next trial if they meet the eligibility criteria, and so on. Eligible individuals can appear as discontinuers in only one trial, but discontinuers may appear in earlier trials as continuers.

### Target trial emulation

#### Time periods

##### Prevalent new-user design

We specify a study eligibility period between 1 January 2016 and 30 June 2018 since exact medication start and stopping dates are available in the UKCFR from 2016 onwards. The end of the eligibility period is chosen to ensure the study follow-up ends before (1) highly effective CFTR modulator therapies became widely prescribed in the UK and (2) the SARS-CoV-2 pandemic affected care pathways and data collection.^23^ Individuals who meet the eligibility criteria within this period will form the base cohort. For comparison, participants were recruited between November 2001 and July 2004 in CF-WISE.^12^

##### Sequential trials design

The same study period between 1 January 2016 and 30 June 2018 is specified, organised into regularly spaced (eg, monthly) artificial trials within that time period. Individuals are eligible for the first trial if they meet all the eligibility criteria (see *Inclusion and exclusion criteria*) on 1 January 2016. This approach is then applied, with updated covariate data where available (at annual reviews), to every successive new time origin between January 2016 and June 2018.

### Time zero

#### Prevalent new-user design

**Figure 4:**
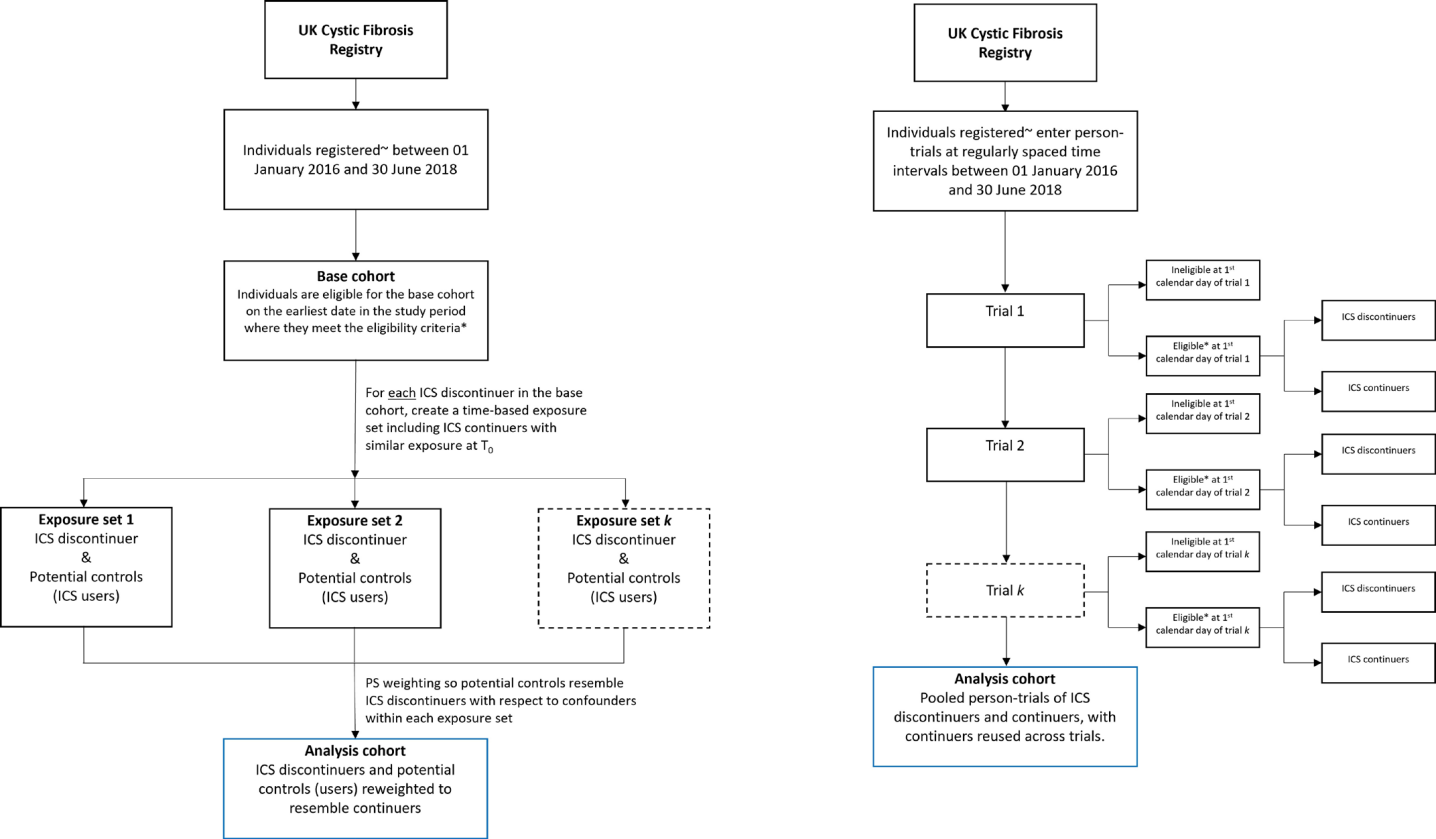
Flow charts for selection of eligible individuals for emulating a target trial of discontinuation of inhaled corticosteroids (ICS) using the UK Cystic Fibrosis Registry (UKCFR), using the prevalent new-user design (PNUD) and sequential trials design. (i) In the PNUD, the first step is formation of a base cohort. The analysis cohort is formed of ICS discontinuers and reweighted comparators who continue ICS. This diagram represents the specifications for the analysis cohort. (ii) In the sequential trials design, individuals enter successive artificial trials at regularly spaced time intervals from new time origins (eg, successive monthly trials with new time origins at the first calendar date of each month in the study period). The ‘trials’ consist of ICS discontinuers and continuers and are analysed as a pooled dataset. T_0_=time zero (date of ICS discontinuation). ~Have a valid annual review in the study period. *Aged ≥6 years, report a FEV_1_ ≥40% predicted (at most recent annual review), using ICS for more than 3 months, no intravenous antibiotics in the 4 weeks prior to time zero.

Time zero in the analysis is equivalent to the time of randomisation in the target trial, defined here as the time at which individuals meet all the eligibility criteria and first discontinue ICS or meet the criteria for being a comparator within an exposure set, thereby entering the emulated trial (see figure 4). In this analysis, due to the availability of exact dates of starting and stopping treatment, ICS continuers are assigned the exact stopping date of the discontinuer as their time zero. This date acts as the time zero for each individual within the exposure set.

#### Sequential trials design

In each trial in the sequential trials design, the time zero will be the stopping date for the ICS discontinuers, and the first day of the calendar time period for that trial for the comparators (those who meet the eligibility criteria and continue on ICS).

### Inclusion and exclusion criteria

Inclusion and exclusion criteria for the index trial, target trial and emulated trial are summarised in table 1.

#### Prevalent new-user design

In the PNUD, to be included in the base cohort individuals should have a diagnosis of CF, be aged ≥6 years, have a forced expiratory volume in 1 s (FEV _1_)≥40% predicted at most recent annual review, and prescribed ICS for >3 months before the date of ICS discontinuation, for both the discontinuer in each exposure set and their corresponding continuers. Excluded are those who were prescribed oral corticosteroids and/or intravenous antibiotics in the 1 month prior to the ICS discontinuation date, for both the discontinuer in each exposure set and their corresponding continuers. Exclusion of individuals taking higher doses of ICS as specified in the target trial is not possible in the emulated trial as dosage information is unavailable in the UKCFR.

#### Sequential trials design

The same inclusion and exclusion will be applied in the sequential trials design, except eligibility will be reassessed based on new information (if an annual review encounter takes place) between each successive trial. The criteria are applied at time zero for each trial, where the index date differs between discontinuers and continuers, unlike in the PNUD.

### Variables

Variable specifications are the same in both the PNUD and sequential trials design.

#### Treatment strategies

The treatment strategies for the TTEs differ from the target trial due to lack of a placebo control. Instead, these analyses consider ICS discontinuation based on prescription data as the ‘treatment’ of interest and continued ICS use as the comparator treatment strategy. Whereas the index trial investigates discontinuation of fluticasone propionate only, the target trial and emulated trials are more pragmatic and will include all types of ICS (including in combination with bronchodilators). This reflects the heterogeneity of specific ICS or IC combination treatments documented within the UKCFR.

Discontinuation will be conceptualised as a point treatment strategy (ie, non-time-varying), where treatment status is determined at a single time point (time zero) for both ICS discontinuers and continuers.

These analyses will first estimate the observational analogue of an intention-to-treat (ITT) effect, which is the effect of being assigned to a treatment status.^24^ We will also target a per-protocol (PP) effect, which is the effect of adhering to the assigned treatment strategies. If in the real world individuals deviate from their assigned treatment (ie, discontinuers switch back to being on ICS and vice versa), the ITT and PP estimands differ and the ITT may be less relevant. See *Main analysis* for details.

#### Outcomes

The primary outcome of the TTE is time to first pulmonary exacerbation. There are multiple definitions of exacerbations in CF clinical studies.^25^ In this TTE, we define a pulmonary exacerbation as a course of intravenous antibiotics given either at home or in hospital, as these treatments are reliably recorded in the UKCFR and have been previously used in landmark studies using observational data in CF.^7 26 27^ This differs from the target trial and CF-WISE, where a symptom-based criteria (known as Fuchs’s definition) was used.^12 28^ The analysis will account for the competing events of death and lung transplant.

Secondary outcomes are change in lung function, measured as change in FEV_1_% predicted and forced vital capacity (FVC%) predicted over 6 months. Spirometry values are calculated based on Global Lung Initiative reference equations-2012.^29^ Change is measured as the absolute difference between measures of FEV_1_% and FVC% measured at baseline (measured at the closest annual review preceding time zero) and at the subsequent annual review, provided that occurs at least 3 months following time zero.

Other secondary outcomes specified in the target trial—new/unplanned courses of intravenous antibiotics and rescue bronchodilator usage—will not be assessed due to similarity of UKCFR definitions for intravenous antibiotic courses and the primary outcome (pulmonary exacerbation) in the TTE, and inability to define ‘rescue’ bronchodilator use using the UKCFR data, respectively.

#### Covariates

In the emulated trials, treatment discontinuation or continuation is not randomised and so measured potential confounders of the relationship between ICS discontinuation and clinical outcomes must be adjusted for to meet the assumption of no unmeasured confounding, a requirement for unbiased causal effect estimation.^30^

Based on a priori clinical expertise, we will adjust for the following covariates at baseline: age, sex, genotype, body mass index z-score, CF-related diabetes, FEV_1_% predicted, home and hospital intravenous antibiotics, hospital admissions, *P. aeruginosa* infection, *Burkholderia* infection, non-tuberculous mycobacteria infection, allergic bronchopulmonary aspergillosis, pancreatic insufficiency, liver disease, inhaled bronchodilator use (captured as a separate variable to ICS), asthmatic status (as a proxy to atopic status specified in CF-WISE), inhaled antibiotic use and mucoactive therapy use (hypertonic saline, mannitol and DNase).

For both the PNUD and sequential trials design, these covariates will be recorded at the nearest annual review date preceding time zero. Since ICS stopping dates may occur at any point between successive annual reviews, stopping dates will occur at various time intervals following baseline annual review across individuals. Therefore, time from previous annual review (in months) to time zero for both the discontinuers and continuers will be adjusted for in the analyses.

### Data analysis

#### Causal estimands

Causal estimands are a way to clearly describe the target of treatment effect estimation.^31^ In an RCT, the average treatment effect in the treated (ATT) and an alternative estimand, the average treatment effect in the whole population (ATE), are the same due to randomised treatment assignment.^24^ In observational data, however, the ATT and the ATE differ because the characteristics of the treated subgroup differ from those of the whole eligible population. In our study, the ATT targets the question: ‘how would the outcomes in those discontinuing ICS have differed, on average, had they continued on ICS?’. By contrast, under the ATE, we ask the question: ‘how would the outcomes in the population differ, on average, had everyone discontinued ICS versus continued on ICS?’.^24^

The PNUD specifically targets an ATT, whereas the sequential trials design, which makes use of all continuers in the study (figure 4) can be used to estimate an ATE or ATT through different analysis steps. This work will establish which estimands are targeted in both study designs, interpret any differences between them and explore how data obtained under the sequential trials design can be used to target the ATT as well as an ATE, and hence allow valid comparison between results obtained under the two designs.

HRs for time to pulmonary exacerbation associated with ICS discontinuation will be the primary causal estimand of interest. HRs are assumed to be constant over time and will allow for empirical benchmarking of the findings to the CF-WISE trial. The marginal risk difference will also be estimated to compare the risk of pulmonary exacerbation associated with discontinuation up to a time horizon of 6 months following treatment assignment.

#### Main analysis

##### Prevalent new-user design

In the PNUD, continuers within each exposure set will be weighted to resemble the discontinuers using propensity score (PS) weighting methods described elsewhere as time-stratified standardised morbidity ratio weighting.^32^,^34^ We will fit a logistic regression model across all exposure sets with treatment discontinuation as the outcome, adjusting for time and the covariates specified in *Covariates*, to estimate the probability of discontinuing versus continuing ICS, deriving weights as follows: discontinuers will receive a weight of 1, and continuers will be weighted by their odds of discontinuing (ie, where PS denotes an individual’s probability of being a discontinuer conditional on their covariate set, and continuers are weighted as PS/(1−PS)).^35^

A weighted Cox proportional hazards model, including treatment status (discontinue versus continue) as the only covariate, will be used to estimate HRs for time to first pulmonary exacerbation, along with bootstrapping used to obtain corresponding 95% CIs. Individuals are censored at the time of death or transplant in this analysis. Marginal cumulative incidence curves will also be estimated under the two treatment strategies. This will be achieved by applying a weighted Aalen-Johansen estimator separately in the discontinuers and the continuers. The analysis takes account of the competing events of death or lung transplant.^36^

The above approach gives an estimate of the ITT effect. Estimation of the PP effect will involve censoring individuals who deviate from their baseline treatment status, which will be accounted for using inverse probability of censoring weighting (IPCW). The probability of remaining uncensored up to a given time will be estimated based on a Cox regression where censoring is the outcome and adjustment is made for the baseline covariates (the same covariates adjusted for in the PS-based weighting procedure). These probabilities will be used to derive IPCW weights to be used in the analysis (weighted Cox proportional hazards model and weighted Aalen-Johansen estimator).^37^

##### Sequential trials design

In the sequential trials design, the sequence of emulated ‘trials’ will be constructed and then pooled (ie, stacked) together for the analysis. Adjusted Cox proportional hazards regression models will be used to estimate conditional HRs, including treatment status at the start of each trial and adjusting for the baseline confounders in each trial.

Conditional HRs (adjusted for baseline covariates) from a cause-specific model for pulmonary exacerbation will be presented. We will also obtain estimates of marginal cumulative incidences based on the cause-specific hazard models for pulmonary exacerbation, and for the competing events of death and transplant, followed by empirical standardisation. Marginal risk differences at 6 months will be obtained from the marginal cumulative incidences. Bootstrapping will be used when estimating all 95% CIs.^38^

As in the PNUD, in addition to the ITT estimate, the PP effect will be estimated by censoring individuals when they deviate from their assigned treatment strategy within each artificial trial. IPCW weights will be derived as specified in the PNUD above, but separately in each artificial trial.

See online supplemental material appendix 1 for additional information on estimands, data analysis and analysis of secondary outcomes.

### Subgroup analyses

Subgroup analyses are the same in both the PNUD and sequential trials design. The authors of CF-WISE were careful in their conclusions, suggesting not all people with CF should discontinue ICS, including those with asthma for which ICS is an established treatment.^12^ In the trial, there was an imbalance in atopic status, with a higher rate in the ICS continuer arm. We will investigate discontinuation effects by asthmatic status, in addition to age (<18 and ≥18 years), baseline lung function (40–70 and >70 FEV_1_% predicted), *P. aeruginosa* infection status, inhaled bronchodilator use (ICS only vs ICS in combination with bronchodilator), provided sample size allows.

### Sensitivity analyses

Sensitivity analyses are planned to be the same in both the PNUD and sequential trials design. We will consider undertaking quantitative bias analysis (QBA) to quantify the potential impact of unmeasured confounding. QBA is an important set of tools for sensitivity analyses that test assumptions and impact of potential biases. One such technique is to use the strength of association between a confounder and outcome among unexposed, the strength of association between the confounder and treatment in the source population and the prevalence of the confounder as bias parameters.^39^

Robustness of results to the definition of the outcome will be assessed using an alternative definition for pulmonary exacerbation, restricting to hospital intravenous antibiotic use (excluding home use) and separately excluding intravenous episodes recorded as ‘planned’.

To address the potential for misclassification or failure to obtain accurate start and stop dates in the UKCFR, we will consider an alternative approach to the study design where an annualised definition of ICS discontinuation collected at annual review (whether the participant has stopped ICS since previous annual review) will be used.

### Missing data

Missing data in covariates will be summarised overall and by treatment status. Patterns of missingness over time will be explored and, based on likely missing data mechanisms, appropriate methods for handling missing data, such as inverse probability weighting and multiple imputation by chained equations, will be considered.^40^

### Patient and public involvement

The James Lind Alliance CF priority setting partnership identified the question ‘What are the effective ways of simplifying the treatment burden of people with CF?’ as a top research priority.^41^ This study addresses this priority by benchmarking an existing RCT and evaluating methods which may be appropriate to inform clinical decision making in an era where discontinuation of long-term treatments in CF is highly topical for the community.

As part of a wider project on TTE in CF, we will work in partnership with people with CF and members of the community through focus group sessions and manuscript review to guide the conduct and dissemination of this study.

### Benchmarking

#### Benchmarking the index trial

Benchmarking is the process for assessing compatibility between the results from a TTE using observational data and an index trial.^42^,^45^ We propose prespecified benchmarking criteria suggested by Wang *et al*,^45^ as follows:

1. Point estimates: Is the direction of the estimated HRs consistent between the trial and the target trial emulation? In addition, do the estimated effects of the emulated trial lie within the 95% CI of CF-WISE? This will be assessed quantitatively by calculating the standardised difference estimate and 95% CI for the difference using bootstrapping.^45^
2. Clinical conclusion: Would the same clinical decision be made based on both the RCT and target trial emulation findings?

The assessment of these two criteria will be made in the context of the achieved harmonisation between the protocol of the index trial, target trial and observational emulation, with particular consideration to the alignment of study populations and causal questions.

#### Comparison of methods

The PNUD and sequential trials design were recently compared under the target trial framework and questions remain around the estimands they target, their suitability to discontinuation questions and model precision in smaller samples.^20^ In addition to benchmarking with CF-WISE, we will compare the point estimates and 95% CIs obtained under both designs. Discussion and exploration of comparisons between the two study designs will also be undertaken, namely with respect to causal estimands, eligibility and cohort definitions, interpretability and computational efficiency.

## Data checks and feasibility analysis

Preliminary inspection of the UKCFR data to address feasibility queries was undertaken. The main objective was to ascertain the number of people registered on the UKCFR who were recorded as discontinuing from ICS in the study period (1 January 2016–30 June 2018). We also ascertained that key variables required for the analysis appear to be reliably recorded.

There were 536 people who discontinued ICS in the study period after at least 90 days of sustained use, before applying the other exclusion criteria (see *Covariates*). ICS start and stop dates appear to have been recorded consistently across the study period. Data on intravenous antibiotic episodes (see *Outcomes*) were also recorded reliably from 2016 onwards with exact dates for the beginning and end of treatment days.

## Limitations

A recent study measured the concordance of TTEs and index RCTs and concluded that close replication is possible but may be difficult to achieve, emphasising the need for principled causal study design.^45^ In particular, benchmarking can be challenging given key differences between the index trial and TTE, including that the study populations may differ. In our context, people from higher socioeconomic backgrounds and with less severe disease are more likely to have participated in the index trial,^46^ and the clinical setting in the CF-WISE trial period (2001–2004) may also differ from that in the TTE period (2016–2018).

The study design relies heavily on accuracy of stop and starting dates for ICS in the UKCFR. Under-reporting or delayed reporting of stopping dates may lead to misclassification of discontinuation. Moreover, ICS use is based on a record of the treatment having been prescribed rather than actual use, and so the medication may have been dispensed but not adhered to.

A key limitation is the potential for unmeasured confounding leading to violation of the conditional exchangeability assumption. While statistical methods in the PNUD and sequential trials design will be used to control for differences in the characteristics of treatment groups with respect to measured covariates, some influence of unmeasured confounders may remain. This limitation will be investigated using QBA methods (*Sensitivity analyses*).

Our definition of the primary outcome may lead to misclassification bias as there are differing thresholds for identifying and treating pulmonary exacerbations in clinical practice, and indications for intravenous antibiotics may differ by CF centres and clinical teams.^25 47^ There are, however, previous studies using this definition to investigate treatment effects using UKCFR data. We will perform a sensitivity analysis to check the robustness of conclusions to the definition for the primary outcome (*Sensitivity analyses*).^26^

Another challenge relating to definitions relates to a diagnosis of asthma in people with CF. Asthma is a key covariate in this study due to asthmatic patients being excluded in CF-WISE. The UKCFR captures asthma as reported by the clinical team. This pragmatic definition does not require specific diagnostic criteria, instead relying on this being documented as a known co-morbidity within the patient’s medical records at site. This is a potential limitation in our study.

## Ethics and dissemination

This study has received approval from the UK CF Registry (UKCFR) Research Committee for both the research and access to data (Data request 531). This study involves human participants, and this work will use anonymised data from the Registry, which has Research Ethics Approval (Reference: 24/EE/0012). Ethical approval has been granted by the LSHTM Ethics Committee (Reference: 31520).

Studies from this project will be submitted for publication in high-impact peer-reviewed journals and presented at international pharmacoepidemiology and CF conferences.

## Supplementary material

10.1136/bmjopen-2025-100894 online supplemental file 1
