# Supplementary material for: Effects of deprescribing from inhaled corticosteroids in people with cystic fibrosis: protocol for a target trial emulation using the UK CF Registry
Source: BMJ Open. 2025 Oct 29;15(10):e100894. doi: 10.1136/bmjopen-2025-100894 (PMC12574348; doi:10.1136/bmjopen-2025-100894)
Supplement: online supplemental file 1 [file bmjopen-15-10-s001.pdf]

Supplementary material

Title: Effect of deprescribing from inhaled corticosteroids in people with cystic fibrosis: protocol for a target trial emulation using the UK CF Registry

Authors

Elliot McClenaghan[1], Julie Rouette[2], Emily Granger[1], Gwyneth Davies[3], Ruth H. Keogh[1], John Tazare[1]

[1] Department of Medical Statistics, London School of Hygiene & Tropical Medicine, Keppel St, London, UK

[2] Global Epidemiology, Office of the Chief Medical Officer GSK R&D, Montreal, Canada

[3] Population, Policy & Practice Dept, UCL Great Ormond Street Institute of Child Health, London, UK

Corresponding author

John Tazare, Department of Medical Statistics, London School of Hygiene & Tropical Medicine, Keppel St, London, UK

john.tazare1@lshtm.ac.uk

Appendix 1: Technical appendix

Causal estimands

In this section we let A represent binary treatment status ($A = 1$ is ICS discontinuation, $A = 0$ is continued ICS use). We let T denote the time to the earliest of the event of interest (pulmonary exacerbation) and the competing composite event of death or transplant, with $D = 1$ denoting the event of interest and $D = 2$ denoting the completing event. The counterfactual event time under treatment strategy $A = a$ ($a = 0{,}1$) is denoted T^a, and the corresponding counterfactual event type is D^a.

The hazard ratio comparing the hazards of pulmonary exacerbation under the strategies of ICS discontinuation versus continuation in the discontinuers can be expressed as:

$$HR_{\{ATT\}} = \frac{h_{1,T^1}(t|A=1)}{h_{1,T^0}(t|A=1)}$$

where $h_{1,T^a}(t|A=1)$ denotes the counterfactual hazard for the event of interest (pulmonary exacerbation, $D = 1$) at time t under treatment strategy $A = a$, among those observed to have $A = 1$. (i.e. among the discontinuers). The marginal risk difference at time t for the ATT is also of interest:

$$RD_{ATT(t)} = \Pr(T^1 \leq t, D^1 = 1|A=1) - \Pr(T^0 \leq t, D^0 = 1|A=1)$$

We will estimate the risk difference at time $t = 6$ months. In the presence of the competing event of death or transplant the risk difference can be interpreted as a total effect of treatment in the treated on the cumulative incidence of pulmonary exacerbation when the competing event is not eliminated, which has been referred to as a 'total effect'.[1]

Main analysis: Prevalent new-user design

The propensity score weighting approach to be used for the PNUD approach involves two stages. First, we estimate propensity scores (PS); defined as the probability of discontinuation conditional on baseline characteristics:

$$PS = P(A = 1|\boldsymbol{L} = \boldsymbol{l})$$

where $\boldsymbol{L}$ represents a sufficient set of baseline covariates to mitigate confounding. The PS will be estimated using logistic regression using the baseline covariates specified in *Section 3.5.3*. The second stage is to derive weights: assigning a weight of 1 to discontinuing individuals and weighting comparators by the odds of probability of discontinuing (PS/(1-PS).[2,3] As noted in the main text, using PS weights of this form has been referred standardised morbidity ratio (SMR) weighting. Similarly to inverse probability of treatment weighting (IPTW), the weighting procedure can be vulnerable to extreme weights. We will therefore consider truncation of weights if large weights are observed.[3]

The analysis is then undertaken as specified in *Section 3.5.3*.

Analysis of secondary outcome (change in lung function)

Analysis of change in lung function (FEV_1 and FVC % predicted) will require a different analytical approach than the primary outcome as it is as it is continuous outcome rather than a time-to-event outcome. Baseline lung function will be recorded at the closest annual review preceding time zero. This value will be assumed to be recorded at time zero. Follow-up lung function will be assessed at subsequent annual review which could be 3 to 18 months following treatment assignment depending on the individual. Change in lung function at 6 months following time zero will then be extrapolated using the baseline and follow-up values. Individuals with no annual review or no lung function recording in the 3-18 months following time zero will be recorded as missing. Missingness in the outcome will be addressed using appropriate statistical methods such as imputation or weighting procedures.

For the secondary outcome of change in lung function between the discontinuers and continuers the causal estimand targeted will be the causal mean difference in the discontinuers, defined as

$$ATT = E\{Y_1(1) - Y_0(1)|A = 1\} - E\{Y_1(0) - Y_0(0)|A = 1\}$$
$$= E\{Y_1(1)|A = 1\} - E\{Y_1(0)|A = 1\}$$

where Y_1 is lung function at follow-up and Y_0 is the lung function at baseline, and $Y_1(a)$ and $Y_0(a)$denote the corresponding counterfactual outcomes under treatment $A = a$.$Y_0(1)Y_0(0)$ and $Y_0(0)$ are equal because treatment cannot affect the baseline outcome measurement.

Estimation of the ATT under the PNUD is then by using a weighted linear regression, with weights the same as for the primary outcome. The linear regression has follow-up lung function as the outcome Y_1, and explanatory variables treatment A, baseline lung function Y_0, time t and an interaction between treatment and time. Time is the time between baseline lung function and follow-up lung function. We will set time to 6 to obtain the final estimates of change in lung function at 6 months. Under the sequential trials design, the ATT will be estimated by fitting a linear regression model for Y_1 including explanatory variables and the set of measured confounders L . The ATT is then estimated as the sum of the coefficients for A and $A \times t$.

References

1. Young JG, Stensrud MJ, Tchetgen Tchetgen EJ, Hernán MA. A causal framework for classical statistical estimands in failure-time settings with competing events. *Statistics in Medicine*. 2020;39(8):1199-1236. doi:10.1002/sim.8471
2. Brookhart MA, Wyss R, Layton JB, Stürmer T. Propensity Score Methods for Confounding Control in Nonexperimental Research. *Circulation: Cardiovascular Quality and Outcomes*. 2013;6(5):604-611. doi:10.1161/circoutcomes.113.000359
3. Desai RJ, Franklin JM. Alternative approaches for confounding adjustment in observational studies using weighting based on the propensity score: a primer for practitioners. *BMJ*. 2019:l5657. doi:10.1136/bmj.l5657
