# Supplementary material for: Effects of deprescribing from inhaled corticosteroids in people with cystic fibrosis: protocol for a target trial emulation using the UK CF Registry
Source: BMJ Open. 2025 Oct 29;15(10):e100894. doi: 10.1136/bmjopen-2025-100894 (PMC12574348; doi:10.1136/bmjopen-2025-100894)
Supplement: Supplementary file 2 [file bmjopen-2025-100894.reviewer_comments.pdf]

PEER REVIEW HISTORY

BMJ Open publishes all reviews undertaken for accepted manuscripts. Reviewers are asked to complete a checklist review form and are provided with free text boxes to elaborate on their assessment. These free text comments are reproduced below.

ARTICLE DETAILS

Title (Provisional)

Effects of deprescribing from inhaled corticosteroids in people with cystic fibrosis: protocol for a target trial emulation using the UK CF Registry

Authors

McClenaghan, Elliot; Rouette, Julie; Granger, Emily; Davies, Gwyneth; Keogh, Ruth; Tazare, John

---

VERSION 1 - REVIEW

**Reviewer**	**1**
**Name**	**Gambazza, Simone**
**Affiliation**	**Università degli Studi di Milano, Clinical Sciences and Community Health**
**Date**	**19-Jun-2025**
**COI**	**None**

I read your study protocol with great interest and would like to commend you on your thoughtful and methodologically sound approach. I have a few comments that I hope might be helpful in further improving the clarity of the study.

1. ICS and Bronchodilator Use

Could you please clarify which variables are available in the registry regarding inhaled therapies? Specifically, are both inhaled corticosteroids (ICS) and inhaled bronchodilators recorded? If so, does this allow you to distinguish between people using ICS alone versus those using ICS in combination with bronchodilators? This distinction would be valuable in assessing treatment discontinuation patterns and in clarifying the specific phenomenon being investigated.

2. Definition of Asthmatic Status

How is "asthmatic status" defined and collected in the Registry? As you know, diagnosing asthma in people with CF is complex due to potential CF-asthma overlap or the presence of asthma-like features. It would be helpful to clarify what criteria were used to classify

asthmatic status in your study, especially considering the somewhat arbitrary definitions used in previous studies such as CFWISE.

Moreover, in terms of potential unmeasured confounders, while I agree with your inclusion of nontuberculous mycobacteria (NTM), I wonder why allergic bronchopulmonary aspergillosis (ABPA) was not considered. Incorporating a directed acyclic graph might help illustrate the clinical reasoning and improve transparency around confounder selection.

Additionally, under "mucoactive therapy," could you confirm whether treatments such as hypertonic saline, mannitol, and rhDNase are included?

3. Subgroup Analyses

Defining asthmatic status will also be crucial for interpreting any subgroup analyses. I also suggest reconsidering the broad age group of <18 years; there may be value in further stratification, if sample size allows. Similarly, including subgroups for patients with NTM and ABPA could enhance the analysis, though I understand this may be limited by sample size.

4. Sample Size and Power

Finally, I would appreciate the inclusion of an a priori sample size calculation or an estimation of anticipated power to better understand the validity.

I hope these comments are constructive and contribute to the further development of your important work. I look forward to seeing the results of your study.

VERSION 1 - AUTHOR RESPONSE

Reviewer 1 comments:	
**I read your study protocol with great interest and would like to commend you on your thoughtful and methodologically sound approach. I have a few comments that I hope might be helpful in further improving the clarity of the study.**	Many thanks for this encouraging feedback on our planned study – it is greatly appreciated.

**1. ICS and Bronchodilator Use**<br>**Could you please clarify which variables are available in the registry regarding inhaled therapies? Specifically, are both inhaled corticosteroids (ICS) and inhaled bronchodilators recorded? If so, does this allow you to distinguish between people using ICS alone versus those using ICS in combination with bronchodilators? This distinction would be valuable in assessing treatment discontinuation patterns and in clarifying the specific phenomenon being investigated.**	Thanks for seeking this important clarification. The Registry does contain separate variables for ICS use and bronchodilator use. Therefore, we can distinguish between people using ICS along versus in combination with bronchodilators. This will be explored and described in our assessment of treatment discontinuation patterns, given a key difference between the CF-WISE trial and our observational emulation is that we will include patients who discontinue ICS while also taking bronchodilators in combination.<br>We have added a clarification on capture of these two variables (**line 329**), and also included an additional subgroup analysis given this key difference between CF-WISE and our emulation (**line 406**).
**2. Definition of Asthmatic Status**<br>**How is "asthmatic status" defined and collected in the Registry? As you know, diagnosing asthma in people with CF is complex due to potential CF-asthma overlap or the presence of asthma-like features. It would be helpful to clarify what criteria were used to classify asthmatic status in your study, especially considering the somewhat arbitrary definitions used in previous studies such as CFWISE.**<br>**Moreover, in terms of potential unmeasured confounders, while I agree with your inclusion of nontuberculous mycobacteria (NTM), I wonder why allergic bronchopulmonary aspergillosis (ABPA) was not considered. Incorporating a directed acyclic graph might help illustrate the clinical reasoning and improve transparency around confounder selection.**<br>**Additionally, under "mucoactive therapy," could you confirm whether treatments such as hypertonic saline, mannitol, and rhDNase are included?**	*Asthmatic status*<br>You are correct to emphasise asthma as an important clinical consideration in our study design and the underlying research question. As this is a registry study, we are limited by the available data variables within the UK CF Registry, and the recording of 'asthma' does not require objective testing to confirm diagnosis. Asthma recorded in the Registry is therefore defined as diagnoses of asthma as reported by the clinical team, and the Registry do not require any specific criteria. We have added an additional paragraph in the Limitations section acknowledging the challenges of diagnosing asthma in people with CF (**line 493**).<br><br>*ABPA*<br>Many thanks – we agree this may be an important consideration and have therefore now included ABPA as a confounder (**line 330**). We decided not to include a DAG, but in line with VanderWeele's paper "Principles of confounder selection"[1], all variables selected for adjustment are reliably collected, defined prior to exposure and plausibly related to treatment (discontinuation) and outcome. We had no concerns around structural selection bias in this setting, and so decided a DAG was not required.<br><br>*Mucoactive therapy*<br>We can confirm that hypertonic saline, mannitol and DNase are captured in the Registry and will be adjusted for separately. This is now clarified in the text in **line 332**.

**3. Subgroup Analyses**<br>**Defining asthmatic status will also be crucial for interpreting any subgroup analyses. I also suggest reconsidering the broad age group of <18 years; there may be value in further stratification, if sample size allows. Similarly, including subgroups for patients with NTM and ABPA could enhance the analysis, though I understand this may be limited by sample size.**	Thank you for these suggestions. Our approach to specifying additional subgroup analyses is as follows: If there are differences between CF-WISE and our target trial emulation results, one reason could be treatment effect heterogeneity by variables with different distributions in CF-WISE and our analysis cohort. If this is the case, and where feasible with respect to sample size, we will consider subgroup analyses, such as by the suggested variables.
**4. Sample Size and Power**<br>**Finally, I would appreciate the inclusion of an a priori sample size calculation or an estimation of anticipated power to better understand the validity.**	Thank you for the opportunity to clarify our rationale for not including a sample size calculation.<br><br>Our primary focus is on interpreting the findings of this analysis against those of the CF-WISE trial. The proposed benchmarking approach, described in **Section 3.8.1**, is consistent with key recent initiatives and commentary on the benchmarking of randomised trials and observational studies, e.g. RCT-DUPLICATE. **[2-4]**<br><br>There are also key features of our emulation which pose challenges for specifying an appropriate sample size calculation. Firstly, our index trial is a non-inferiority trial rather than a superiority trial and the primary focus is not on detecting a hazard ratio on a given magnitude. Secondly, as described above, our real interest is whether the emulation gives a similar result to the CF-WISE index trial, and any sample size calculation should be based on that. However, guidance on how to appropriately make these calculations is lacking, ignoring for additional complexities due to the index trial being a non-inferiority trial.<br><br>More generally, our observational analysis relies on historical data that is already collected and will include all eligible individuals within the dataset. Traditional sample size considerations for enrolment are less relevant in this setting, e.g. since we are unable to influence enrolment for the study. Instead, the central goal of a valid causal analysis of observational data such as ours is to produce an internally valid estimate (regardless of its precision) that can be strengthened through replication in similar studies and where appropriate, combined in a meta-analysis.[5]

References

1. Vanderweele TJ. Principles of confounder selection. European Journal of Epidemiology. 2019;34(3):211-9. doi: 10.1007/s10654-019-00494-6.
2. Franklin JM, Patorno E, Desai RJ, Glynn RJ, Martin D, Quinto K, et al. Emulating Randomized Clinical Trials With Nonrandomized Real-World Evidence Studies. Circulation. 2021;143(10):1002-13. doi: 10.1161/circulationaha.120.051718.
3. Dahabreh IJ, Robins JM, Hernán MA. Benchmarking Observational Methods by Comparing Randomized Trials and Their Emulations. Epidemiology. 2020;31(5):614-9. doi: 10.1097/ede.0000000000001231.
4. Matthews AA, Dahabreh IJ, Fröbert O, Lindahl B, James S, Feychting M, et al. Benchmarking observational analyses before using them to address questions trials do not answer: an application to coronary thrombus aspiration. American Journal of Epidemiology. 2022. doi: 10.1093/aje/kwac098.
5. Hernán MA. Causal analyses of existing databases: no power calculations required. Journal of Clinical Epidemiology. 2022;144:203-5. doi: 10.1016/j.jclinepi.2021.08.028.

VERSION 2 - REVIEW

**Reviewer**	**1**
**Name**	**Gambazza, Simone**
**Affiliation**	**Università degli Studi di Milano, Clinical Sciences and Community Health**
**Date**	**26-Sep-2025**
**COI**	

Dear Authors, thank you for your clarifications.
